# Effect of microcapsules of chia oil on Ω-3 fatty acids, antioxidant characteristics and oxidative stability of butter

**DOI:** 10.1186/s12944-020-1190-5

**Published:** 2020-01-16

**Authors:** Rahman Ullah, Muhammad Nadeem, Muhammad Imran, Muhammad Kamran Khan, Zarina Mushtaq, Muhammad Asif, Ahmad Din

**Affiliations:** 1 Department of Dairy Technology, University of Veterinary and Animal Sciences, Outfall Road, Lahore, Pakistan; 2 Institute of Home and Food Sciences, Faculty of Life Sciences, Government College University, Faisalabad, Pakistan; 3 Planning and Development Division, Pakistan Agricultural Research Council, Islamabad, Pakistan; 4 Postharvest Research Station, Ayub Agricultural Research Institute, Faisalabad, Pakistan

**Keywords:** Microencapsulation, Chia oil, Antioxidant capacity, Oxidative stability, Butter

## Abstract

**Background:**

Ω-3 fatty acids perform several therapeutic functions in the body, however, their applications are limited due to the inferior oxidative stability. To improve the oxidative stability and release properties of Ω-3 fatty acids, microencapsulation is performed. Butter is a good source of fat-soluble vitamins and antioxidant systems however, it is not a good source of Ω-3 fatty acids. Supplementation of butter with microcapsules of vegetable oils rich in Ω-3 fatty acids is not reported in literature.

**Methods:**

Microcapsules of chia oil (MCO) were prepared using chitosan as encapsulating material by spray drying at lower temperature. Unsalted butter prepared from cultured cream using *Lactococcus lactis* ssp. *Lactis* at 21 °C for 16 Hrs. Cream was churned at 12 °C and microcapsules of chia oil were added to the butter during the working stage at four different concentrations i.e. 2, 4, 6 and 8% (T_1_, T_2_, T_3_ and T_4_, respectively). Butter without supplementation of MCO were kept as control. Butter samples were stored for 90 days at -10 °C. Butter composition, antioxidant capacity, fatty acid profile, induction period, free fatty acids, peroxide value and sensory evaluation were performed at 0, 45 and 90 days of storage.

**Results:**

Addition of MCO in butter did not have any effect on standards of identity of butter. Microencapsulation had no effect on fatty acid profile of microcapsules of chia oil. Concentration of alpha-linolenic acid (ALA) in control, T_1_, T_2_, T_3_ and T_4_ were 0.49, 4.29, 8.41, 13.21 and 17.44%, respectively. Concentration of ALA in fresh and 90 days stored butter samples were 17.44 and 17.11%, respectively. After 90 days of storage, loss of eicosapentaenoic acid (EPA), docosapentaenoic acid (DPA) and docosahexaenoic acid (DHA) were 0.07%, 0.05 and 0.03%, respectively. At 0, 45 and 90 days of storage, 2, 2-Diphenyl-1-picrylhydrazyle (DPPH) free radical scavenging activity of free chia oil was 39.81, 71.22 and 62.18%, respectively. However, microcapsules of chia oil had superior antioxidant activity. DPPH free radical scavenging activity of microcapsules at 0, 45 and 90 days of storage was 36.51, 36.43 and 35.96%, respectively (*p* > 0.05). Total antioxidant capacity of microcapsules at 0, 45 and 90 days of storage was 70.53, 69.88 and 68.52%, respectively (*p* > 0.05). It was recorded that induction period of free chia oil and microcapsules was only 2.86 h and 8.55 h. Among the butter samples, control revealed the lowest induction period. While, induction period of experimental samples was not different from each other. Peroxide value and free fatty acids of the butter samples at the end of storage period (90 days) was less than the European Union standards limit (10MeqO_2_/kg and 0.2%). Sensory characteristics of experimental samples were similar to the control. MCO can be added in butter to improve its functional value.

**Conclusion:**

Concentration of Ω-3 fatty acids in butter up to 8% can be increased through microcapsules of chia oil with reasonable oxidative stability and no effect on sensory characteristics.

## Background

The increased knowledge of metabolic disorders caused by the foods has led to use functional foods. In the last decade, great deal of research work has been done on the development and commercialization of functional foods and ingredients [1]. A noteworthy increase in the consumption of functional foods containing polyunsaturated fatty acids [2]. Cardiovascular diseases and cancer are one of the largest killers of mankind. About 200,000 people die every year due to cardiovascular diseases [3, 4]. Metabolic diseases can be prevented by the regular intake of functional foods. Dairy Products are the integral part of human nutrition, it contain some Ω-3 fatty acids, but the concentration is usually lower than the biologically required values. Alpha-linolenic acid (ALA), eicosapentaenoic acid (EPA) and docosahexaenoic acid (DHA) are the most important Ω-3 fatty acids from therapeutic point of view [5]. These fatty acids have several health promising effects such as cardio-protective, anti-stressing, anti-ageing, anti-inflammatory and antidiabetic properties [6]. In growing babies, Ω-3 fatty acids help in the development of brain and cardiovascular system and cardio-protective effects [7]. Due to the better absorption of omega-3 fatty acids of fish origin, it is regarded as a great source of Ω-3, however, fish alone cannot fulfill the dietary requirement of Ω-3 fatty acids [8]. Due to the persistence of fishy flavor in fish oil, it has limited applications for the fortification strategies [9]. FDA recommends to consume 2 g Ω-3 fatty acids on daily basis, to minimize the incidences of cardiovascular diseases. To fulfill the dietary requirements of Ω-3 fatty acids, foods containing Ω-3 fatty acids are being developed all over the world. Demand of functional foods supplemented with Ω-3 fatty acids is increasing across the globe. According to the report of Northwestern Regional Project, cultivated area of chia seed in 13 countries was 370,000 ha. Presently, chocolates, biscuits, bread, yoghurt and ice cream are being supplemented with Ω-3 fatty acids [10]. New sources of Ω-3 fatty acids should be discovered for increased industrial utilization. Chia (*Salvia hispanica* L.) produces about 35–40% superior quality edible oil and it contains about 60–65% ALA oil which can be used for the supplementation of foods without pre-processing, such as refining, bleaching and deodorization [11]. Chia has obtained the status of Novel Food from the EU parliament with no anti-nutritional factors [12]. For example, fish contains about 14% Ω-3 fatty acids, while, chia oil contains about 60–65% ALA. Direct addition of Ω-3 rich oils in foods is technically challenging due to their susceptibility towards auto-oxidation, objectionable odors and incompatibility with food matrixes [13] and even more, oxidation of C_18:1_, C_18:2_ and C_18:3_ has been found greater than C_18:0_ [5, 14]. Oxidative deterioration in fat rich foods reduces the nutritional value and sensory characteristics of foods [15]. Nadeem et al. [16] increased the concentration of ALA in *trans* free margarine through direct addition of chia oil and the resulted margarine had higher magnitude of Ω-3 fatty acids but the oxidative stability of chia oil supplemented margarine was lower than the market margarine. Ice cream supplemented with olein fraction of chia oil had higher amount of ALA with lower oxidative stability than the standard ice cream [17]. Oils rich in polyunsaturated fatty acids (PUFAs) are commercially preserved by synthetic antioxidants, which may be carcinogenic [18]. Microencapsulation is a process in which bioactive compounds are enclosed, shielded and sheltered with a polymeric film such as modified starch, cylodextrins, cellulose, chitosan, gum Arabic, caseinates, whey proteins and lecithin [19]. Microcapsules may be prepared by spray drying, centrifugal extrusion etc. however, spray drying is the most commonly used method for the preparation of microcapsules of bioactive compounds for the fortification in foods [20]. Another important reason for the microencapsulation is to regulate the release characteristics of oils rich in Ω-3 fatty acids at the suitable time and place with increased effectiveness at the lower concentrations [21]. For the preparation of microcapsules in food products, spray drying is the most commonly used technique as the anticipated protection, release profile and ease of large-scale production [22]. Studies of Ullah et al. [17] showed that chia oil can be used in dairy products, however, no study is available regarding the addition of microcapsules of chia oil to the butter. Therefore, this study was planned to raise the content of Ω-3 fatty acids in butter by microencapsulated chia oil and study the oxidative stability, antioxidant characteristics and sensory characteristics of fortified butter by some conventional and advanced analytical techniques.

## Methods

### Materials

Chitosan 78 was purchased from Sigma Aldrich; chia oil from unheated seeds was obtained by a laboratory scale expeller. Butter without any addition of salt was obtained from a local dairy industry.

### Preparation of microcapsules of chia oil

For the preparation of microcapsules of chia oil, chitosan was used as encapsulating material. Chitosan was dissolved at 5% concentration in 1% aqueous acetic acid, oil was added at the rate of 50% by weight of chitosan, homogenized at 200 Bar. Emulsion were spray dried in a mini spray dryer, size of the nozzle was 0.4 mm with 0.55 MPa air pressure. Temperature of the air was set at 160 °C and outlet temperature was 90 °C. Microcapsules were packaged in transparent plastic bottles and store at -10 °C till further usage in current investigation.

### Experimental plan

This research work was planned in a completely randomized design, every treatment was replicated three times. Unsalted butter prepared from cultured cream using *Lactococcus lactis* ssp. *Lactis* as starter culture at 21 °C for 16 Hrs. Cream was churned at 12 °C and microcapsules of chia oil were added to the butter during the working stage at four different concentrations i.e. 2% (T_1_), 4% (T_2_), 6% (T_3_) and 8% (T_4_), respectively. Butter samples without supplementation of MCO were regarded as control. Butter samples were stored for 90 days at -10 °C and analyzed at 0, 45 and 90 days of storage.

### Chemical composition of butter

Fat, moisture and non-fat dry matter contents of freshly prepared control and experimental butter samples were analyzed as per reference methods [23]. Free fatty acids, peroxide value, melting point, saponification value, unsaponifiable matter and iodine value (Wijs) in cold pressed chia oil were determined according to the standard methods [24].

### Antioxidant activity of free oil, microcapsules and butter

#### DPPH free radical scavenging activity

Total antioxidant activity of free oil, microcapsules and butter were determined according to the method [24]. Samples were added with 4 mL DPPH solution (0.1 M) followed by vigorous shaking and incubation for 30 min at room temperature. The absorbance was measured at 517 nm in visible regions of spectrum. Same concentration of tertiary butylated hydroxy Quinone was used as reference and samples were analyzed on 0, 45 and 90 days of storage.

#### Total antioxidant activity

Total antioxidant activity of free chia oil, microcapsules and butter samples was determined using phosphomolybdenum method [25]. Sample solution 0.3 ml (100 μg/mL) was mixed with 3 ml sulfuric acid, (0.6 M) and sodium phosphate (28 mM) and ammonium molybdate (4 mM). The tubes were capped and incubated in water bath maintained at 95 °C for a time duration of 90 min. Samples were cooled down to room temperature and absorbance were recorded at 695 nm on a spectrophotometer. Total antioxidant capacity was expressed in terms of mg Ascorbic Acid Equivalent/g and samples were analyzed on 0, 45 and 90 days of storage.

### Fatty acid profile

Fat from microcapsules of chia oil and butter was extracted by following the standard method [23]. One drop of extract fat/ oil (50 mg) was taken in 15 mL Pyrex test tube, 2 ml methanolic hydrogen chloride (15%) was added, test tubes were tightly capped and put in the heating block in a fume hood. After 100 min, tubes were let to cool to room temperature, then 2 mL each n-hexane and deionized water were added, followed by vertexing at 1500 rpm for 3 min, after 30 min, upper layer was used for GC analysis (7890-B, Agilent Technologies) fitted with FID, using ZB-5 fused silica capillary column (Zebron Phenomenex, 100 m × 0.250 mm) using helium, hydrogen and oxygen at the rate of 1.2 mL/min, 4 mL/min and 40 mL/min, with a total run time was 43 min and samples were analyzed on 0, 45 and 90 days of storage. FAME-37 (Supleco) standard was run for the identification and quantification of fatty acids [26].

### Induction period

Induction period was determined using Rancimat technique (Professional Rancimat, 892), for this analysis, 2.5 g sample was exposed to dry oxygen (20 l/hr) at 120 °C. Break point in the curve was used to denote the induction period on 0, 45 and 90 days of storage period (Metrohm Corporation, Switzerland).

### Storage effect on oxidative stability of butter

MCO supplemented butter samples and control were analyzed for free fatty acids, peroxide value and anisidine value on 45 and 90 days of storage [24].

### Sensory evaluation

MCO supplemented butter samples were tested for color, oxidized flavor and texture in individual sensory evaluation booths on 0, 45 and 90 days of storage. Ten trained judges performed the sensory evaluation using 9-point scale [27].

### Statistical analysis

This experiment was planned in a completely randomized design, treatment was replicated three times and every sample was analysed three times, data were reported as Mean ± SD. Data were analysed by two way analysis of variance technique to determine the effect of treatments, storage and their interaction. To determine the significant difference among the treatments, Duncan Multiple Range Test was used on a SAS 9.1 software [28].

## Results

Free fatty acids, peroxide value, saponification value, unsaponifiable matter, slip melting point and iodine value (wijs) of chia oil used in this investigation were 0.11%, 0.23 (MeqO_2_/kg), 191 mg KOH/g, 1.28%, − 17.6 °C and 192cg/100 g, respectively. Outcomes of composition of butter supplemented with Ω-3 powder of chia oil are presented in Table 1. Fat, moisture content (MC) and non-fat dry matter content (NFDMC) of butter were influenced by the supplementation of Ω-powder (*p* < 0.05). Fat, NFDMC of the butter increased while MC decreased as a function of supplementation of microcapsules.

**Table 1:** Effect of microencapsulated chia oil on composition of butter

Treatments	Fat (%)	Moisture (%)	NFDMC (%)
Control	83.4 ± 0.75^c^	15.19 ± 0.05^a^	1.41 ± 0.11^d^
T_1_	83.9 ± 1.12^c^	14.58 ± 0.03^a^	1.52 ± 0.08^c^
T_2_	85.1 ± 0.94^b^	13.13 ± 0.29^b^	1.77 ± 0.12^b^
T_3_	85.8 ± 0.65^b^	12.39 ± 0.15^c^	1.81 ± 0.19^a^
T_4_	87.5 ± 0.43^a^	10.58 ± 0.17^d^	1.92 ± 0.13^a^

In a column, means notified by a non-uniform letter are statistically significant (*p* < 0.05)

NFDMC: Not Fat Dry Matter Content

Control: Butter without any addition of Ω-3 powder

T_1_: Butter with 2% addition of microcapsules chia oil

T_2_: Butter with 4% addition of microcapsules chia oil

T_3_: Butter with 6% addition of microcapsules chia oil

T_4_: Butter with 8% addition of microcapsules chia oil

It was recorded that antioxidant properties of spray dried microcapsules of chia oil was not different from free chia oil (Table 2). The effect of storage duration of 90 days on antioxidant characteristics of free oil and microcapsules were also investigated. It was recorded that DPPH free radical scavenging activity and total antioxidant capacity of free chia oil was remarkably affected by the storage interval of 90 days. Microcapsules of chia oil had superior antioxidant activity. DPPH free radical scavenging activity of microcapsules at 0, 45 and 90 days of storage was 36.51, 36.43 and 35.96%, respectively (*p* > 0.05). Total antioxidant capacity of microcapsules at 0, 45 and 90 days of storage was 70.53, 69.88 and 68.52%, respectively (*p* > 0.05). It was recorded that microcapsules of chia oil raised the antioxidant capacity of butter in a dose dependent manner. Butter samples supplemented with 8% concentration of microcapsules of chia oil exhibited the highest antioxidant capacity. DPPH free radical scavenging activity and total antioxidant capacity of T_4_ was 27.79 and 55.93%. DPPH free radical scavenging activity and total antioxidant capacity of all the treatments of butter were not affected by the storage period of 90 days.

**Table 2:** Antioxidant activity of free oil, microcapsules and butter

Treatments	Storage Days	DPPH Free Radical Scavenging Activity	Total Antioxidant Capacity
Free Oil	0	39.81 ± 0.27^a^	73.61 ± 0.40^a^
	45	33.23 ± 0.22^c^	71.22 ± 0.36^b^
	90	24.62 ± 0.16^e^	62.18 ± 0.12^d^
MCO	0	38.51 ± 0.18^a^	73.53 ± 0.55^a^
	45	38.43 ± 0.23^a^	73.44 ± 0.72^a^
	90	35.96 ± 0.15^b^	68.52 ± 0.16^c^
sControl	0	14.69 ± 0.08^h^	30.75 ± 0.26^i^
	45	11.19 ± 0.05^i^	30.18 ± 0.62^i^
	90	6.43 ± 0.02^j^	19.66 ± 0.49^j^
T_1_	0	17.53 ± 0.15^g^	35.16 ± 0.05^h^
	45	17.33 ± 0.05^g^	35.11 ± 0.12^h^
	90	16.25 ± 0.06^g^	34.95 ± 0.07^h^
T_2_	0	20.43 ± 0.19^f^	43.77 ± 0.30^g^
	45	20.18 ± 0.25^f^	43.49 ± 0.27^g^
	90	19.77 ± 0.31^f^	43.33 ± 0.22^g^
T_3_	0	25.16 ± 0.44^e^	46.83 ± 0.82^f^
	45	24.89 ± 0.37^e^	46.22 ± 0.73^f^
	90	24.91 ± 0.05^e^	46.10 ± 0.43^f^
T_4_	0	27.79 ± 0.13^d^	55.93 ± 0.38^e^
	45	27.62 ± 0.03^d^	55.61 ± 0.28^e^
	90	27.33 ± 0.22^d^	55.12 ± 0.10^e^

In a column, means notified by a non-uniform letter are statistically significant (*p* < 0.05)

MCO Microcapsules of chia oil stored at room temperature (25-27 °C) for 90 days

**Fig. 1:**
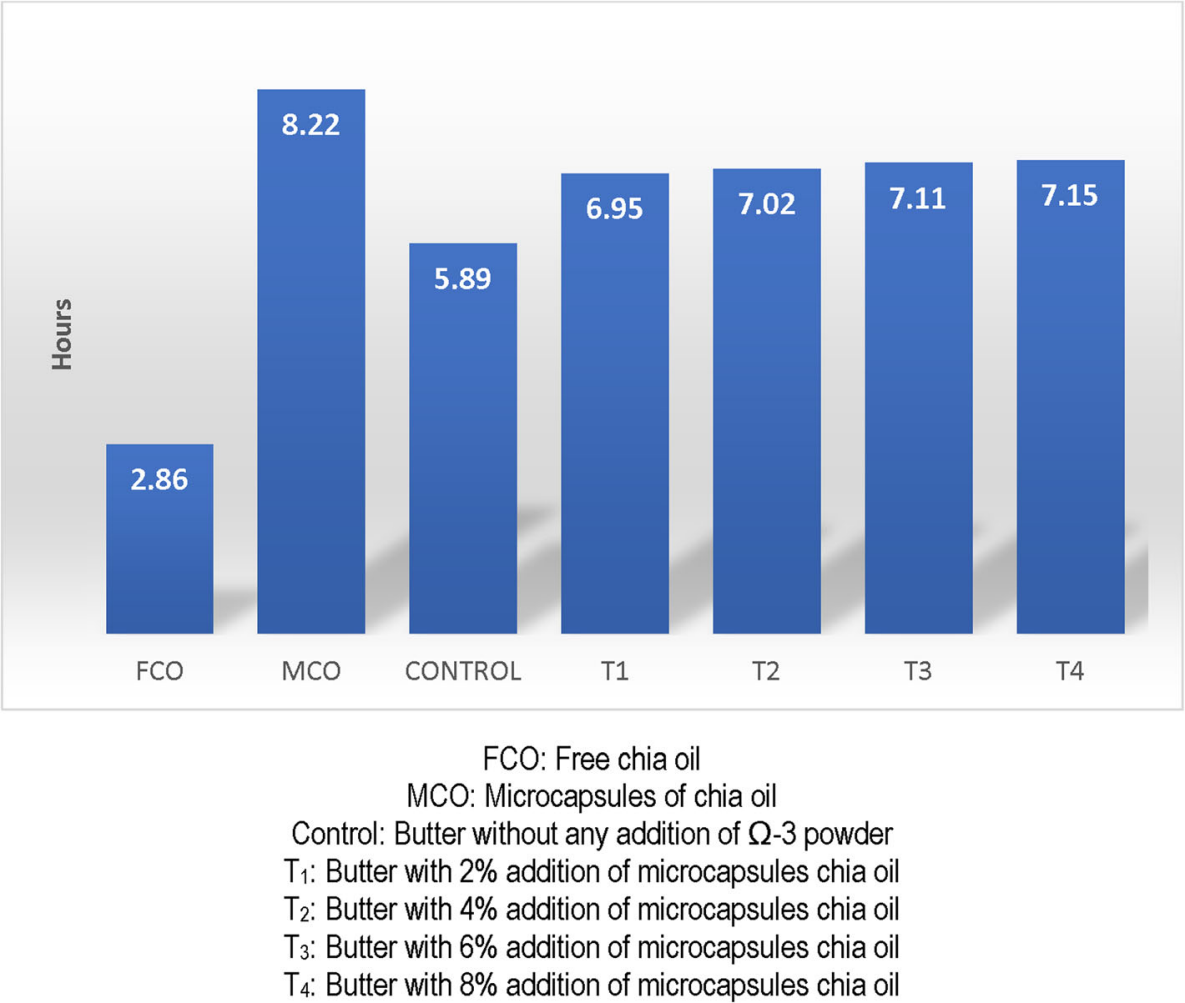
Induction of microencapsulated chia oil supplemented butter

Fatty acid profile of the free and encapsulated forms of chia oil are shown in Table 3. Results of this investigation revealed that fatty acid profile of microcapsules of chia oil was not affected by the spray drying (*p* < 0.05). Supplementation of butter with MCO raised the amount of ALA, EPA and DHA (Table 2). Results in Table 4 showed that concentration of ALA in fresh and 90 days stored butter samples were 17.44 and 17.11%, respectively. After 90 days of storage, loss of EPA and DPA and DHA were 0.07%, 0.05 and 0.03%, respectively. Peroxide value of MCO supplemented butter samples and control amplified during the whole storage period. With respect to peroxide value, storage period up to 45 days was non-significant (Table 5). Peroxide value of T_4_ was within the standard limit (10 meqO_2_/kg). Maximum peroxide value was recorded in T4 (1.41 MeqO_2_/kg) after 90 days of storage. Free fatty acids of mechanically extracted chia oil were 0.08%. No difference was recorded in free fatty acids content of all the samples and control. Anisidine value of MCO supplemented butter samples and control were not changed up to 45 days of storage (*p* < 0.05). For the determination of induction period of free chia oil, microcapsules of chia and supplemented butter samples, Professional Rancimat 832 was used. All the samples were run without fat extraction and the obtained results can be seen in Fig. 1. In this investigation, effect of microencapsulation and storage duration on butter samples supplemented with four different concentrations of microcapsules was determined on induction period. It was recorded that induction period of free chia oil and microcapsules was only 2.86 h and 8.55 h. Among the butter samples, control revealed the lowest induction period. While, induction period of experimental samples was not different from each other. Results regarding sensory properties of MCO supplemented butter are shown in Table 6. Non-significant findings were observed in color, flavor and texture of all the sample and control.

**Table 3:** Effect of microencapsulated chia oil on fatty acid profile of butter

Fatty Acid	Free Chia Oil	MCO*	Control	T_1_	T_2_	T_3_	T_4_
C_4:0_	–	–	1.84 ± 0.01^a^	1.67 ± 0.03^b^	1.51 ± 0.02^c^	1.42 ± 0.06^d^	1.37 ± 0.09^e^
C_6:0_	–	–	2.21 ± 0.06^a^	2.11 ± 0.02^b^	1.97 ± 0.13^c^	1.81 ± 0.05^d^	1.62 ± 0.03^e^
C_8:0_	–	–	2.35 ± 0.03^a^	2.24 ± 0.09^b^	2.12 ± 0.11^c^	1.99 ± 0.04^d^	1.91 ± 0.06^e^
C_10:0_	–	–	2.45 ± 0.09^a^	2.29 ± 0.08^b^	2.15 ± 0.05^c^	1.97 ± 0.04^d^	1.95 ± 0.13^d^
C_12:0_	–	–	2.69 ± 0.10^a^	2.48 ± 0.15^b^	2.25 ± 0.03^c^	2.12 ± 0.06^d^	1.89 ± 0.15^e^
C_14:0_	0.11 ± 0.01^e^	0.12 ± 0.03^e^	10.33 ± 0.19^a^	9.81 ± 0.51^b^	8.66 ± 0.25^c^	8.24 ± 0.18^c^	7.30 ± 0.26^d^
C_16:0_	9.33 ± 0.05^f^	9.36 ± 0.27^f^	31.29 ± 0.41^a^	29.36 ± 0.49^b^	28.26 ± 0.71^c^	27.15 ± 0.39^d^	26.59 ± 0.44^e^
C_18:0_	1.57 ± 0.03^d^	1.59 ± 0.04^d^	9.77 ± 0.15^a^	9.41 ± 0.16^a^	8.66 ± 0.22^b^	8.36 ± 0.17^b^	7.27 ± 0.36^c^
C_18:1_	6.85 ± 0.09^f^	6.88 ± 0.19^f^	24.51 ± 0.31^a^	23.29 ± 0.88^b^	22.19 ± 0.28^c^	21.77 ± 0.35^d^	19.95 ± 0.54^e^
C_18:2_	19.49 ± 0.13^a^	19.55 ± 0.38^a^	4.27 ± 0.08^f^	5.11 ± 0.10^e^	6.22 ± 0.17^d^	7.58 ± 0.19^c^	8.95 ± 0.37^b^
ALA	66.45 ± 0.19^a^	66.72 ± 0.81^a^	0.49 ± 0.02^f^	4.29 ± 0.14^e^	8.41 ± 0.71^d^	13.21 ± 0.31^c^	17.44 ± 0.42^b^
EPA	2.81 ± 0.06^a^	2.82 ± 0.06^a^	Not Detected	0.11 ± 0.02^e^	0.19 ± 0.04^d^	0.35 ± 0.06^c^	0.56 ± 0.05^b^
DPA	1.91 ± 0.08^a^	1.93 ± 0.09^a^	Not Detected	0.09 ± 0.01^e^	0.17 ± 0.06^d^	0.29 ± 0.01^c^	0.41 ± 0.01^b^
DHA	2.36 ± 0.02^a^	2.38 ± 0.05^a^	Not Detected	0.05 ± 0.03^e^	0.21 ± 0.02^d^	0.31 ± 0.04^c^	0.45 ± 0.07^b^

In a row, means bearing a non-common letter are statistically different (*p* < 0.05)

ND: Not Detected

MCO: *Microcapsules of chia oil

ALA = Alpha Linolenic acid

EPA = Eicosapentaenoic acid

DPA = Docosapentaenoic acid

DHA = Docosahexaenoic acid

**Table 4:** Effect of microencapsulated chia oil on fatty acid profile of butter.

Fatty Acid	Control		T_1_		T_2_		T_3_		T_4_	
	Fresh	90-Days Old	Fresh	90-Days Old	Fresh	90-Days Old	Fresh	90-Days Old	Fresh	90-Days Old
C_4:0_	1.84 ± 0.01^a^	1.81 ± 0.02^a^	1.67 ± 0.03^b^	1.65 ± 0.02^b^	1.51 ± 0.02^c^	1.48 ± 0.06^c^	1.42 ± 0.06^d^	1.340 ± 0.01^d^	1.33 ± 0.09^d^	1.31 ± 0.03^d^
C_6:0_	2.21 ± 0.06^a^	2.21 ± 0.03^a^	2.11 ± 0.02^b^	2.08 ± 0.05^b^	1.97 ± 0.13^c^	1.95 ± 0.08^c^	1.81 ± 0.05^d^	1.75 ± 0.04^d^	1.62 ± 0.03^e^	1.57 ± 0.01^e^
C_8:0_	2.35 ± 0.03^a^	2.31 ± 0.04^a^	2.24 ± 0.09^b^	2.21 ± 0.02^b^	2.12 ± 0.11^c^	2.09 ± 0.01^c^	1.99 ± 0.04^d^	1.95 ± 0.03^d^	1.88 ± 0.06^e^	1.86 ± 0.07^e^
C_10:0_	2.45 ± 0.09^a^	2.40 ± 0.09^a^	2.29 ± 0.08^b^	2.27 ± 0.05^b^	2.15 ± 0.05^c^	2.11 ± 0.03^c^	1.97 ± 0.04^d^	1.91 ± 0.02^d^	1.95 ± 0.13^d^	1.92 ± 0.08^d^
C_12:0_	2.69 ± 0.10^a^	2.62 ± 0.05^a^	2.48 ± 0.15^b^	2.46 ± 0.01^b^	2.25 ± 0.03^c^	2.20 ± 0.01^c^	2.12 ± 0.06^d^	2.05 ± 0.07^d^	1.89 ± 0.15^e^	1.81 ± 0.09^e^
C_14:0_	10.33 ± 0.19^a^	10.14 ± 0.36^a^	9.81 ± 0.51^b^	9.55 ± 0.21^b^	8.66 ± 0.25^c^	8.55 ± 0.56^c^	8.51 ± 0.18^c^	8.11 ± 0.28^d^	7.30 ± 0.26^e^	7.19 ± 0.57^e^
C_16:0_	31.29 ± 0.41^a^	30.85 ± 1.19^a^	29.36 ± 0.49^b^	29.11 ± 1.05^b^	28.76 ± 0.71^b^	27.54 ± 0.64^c^	27.15 ± 0.39^c^	26.65 ± 0.72^d^	26.59 ± 0.44^d^	26.40 ± 0.47^d^
C_18:0_	9.77 ± 0.15^a^	9.62 ± 0.17^a^	9.41 ± 0.16^a^	9.27 ± 0.46^a^	8.66 ± 0.22^b^	8.44 ± 0.23^b^	8.36 ± 0.17^b^	8.22 ± 0.09^b^	7.27 ± 0.36^c^	7.16 ± 0.08^c^
C1_8:1_	24.51 ± 0.31^a^	24.19 ± 0.88^a^	23.29 ± 0.88^b^	23.04 ± 0.76^b^	22.19 ± 0.28^c^	22.07 ± 0.62^c^	21.77 ± 0.35^d^	21.51 ± 0.26^d^	19.95 ± 0.54^e^	19.46 ± 0.73^e^
C_18:2_	4.27 ± 0.08^e^	4.19 ± 0.09^e^	5.11 ± 0.10^d^	4.97 ± 0.13^d^	6.22 ± 0.17^c^	6.10 ± 0.08^c^	7.58 ± 0.19^b^	7.44 ± 0.16^b^	8.95 ± 0.37^a^	8.82 ± 0.31^a^
ALA	0.49 ± 0.02^e^	0.45 ± 0.03^e^	4.29 ± 0.14^d^	4.15 ± 0.03^d^	8.41 ± 0.71^c^	8.33 ± 0.22^c^	13.21 ± 0.31^b^	13.07 ± 0.05^b^	17.44 ± 0.42^a^	17.41 ± 0.53^a^
EPA	ND	ND	0.11 ± 0.02^d^	0.08 ± 0.01^d^	0.19 ± 0.04c	0.16 ± 0.02^c^	0.35 ± 0.06^b^	0.30 ± 0.03^b^	0.56 ± 0.05^a^	0.49 ± 0.01^a^
DPA	ND	ND	0.09 ± 0.01^e^	0.07 ± 0.03^e^	0.17 ± 0.06^d^	0.13 ± 0.02^d^	0.29 ± 0.01^b^	0.25 ± 0.03^b^	0.41 ± 0.01^a^	0.36 ± 0.04^a^
DHA	ND	ND	0.05 ± 0.03^d^	0.07 ± 0.02^d^	0.21 ± 0.04^c^	0.19 ± 0.01^c^	0.31 ± 0.04^b^	0.28 ± 0.06^b^	0.45 ± 0.07^b^	0.42 ± 0.09^a^

In a row, if the means are bearing different alphabet, that are statistically significant (p < 0.05)

ND: Not Detected

ALA = Alpha Linolenic acid

EPA = Eicosapentaenoic acid

DPA = Docosapentaenoic acid

DHA = Docosahexaenoic acid

**Table 5:** Oxidative stability of butter supplemented with microencapsulated chia oil

Treatments	Storage Days	FFA (% Oleic Acid)	PV (MeqO_2_/Kg)	Anisidine Value
Control	0	0.08 ± 0.01^c^	0.22 ± 0.03^f^	4.51 ± 0.16^f^
	45	0.11 ± 0.02^b^	0.38 ± 0.04^e^	4.89 ± 0.07^e^
	90	0.14 ± 0.03^a^	0.59 ± 0.07^d^	7.95 ± 0.23^d^
T_1_	0	0.08 ± 0.02^c^	0.22 ± 0.03^f^	4.51 ± 0.16^f^
	45	0.11 ± 0.03^b^	0.41 ± 0.06^e^	4.95 ± 0.15^e^
	90	0.13 ± 0.02^a^	0.69 ± 0.05^d^	8.55 ± 0.09^c^
T_2_	0	0.08 ± 0.03^c^	0.22 ± 0.03^f^	4.51 ± 0.16^f^
	45	0.10 ± 0.01^b^	0.45 ± 0.02^e^	5.11 ± 0.29^e^
	90	0.14 ± 0.02^a^	0.82 ± 0.10^c^	9.13 ± 0.41^b^
T_3_	0	0.08 ± 0.01^c^	0.22 ± 0.03^f^	4.51 ± 0.16^f^
	45	0.12 ± 0.02^a^	0.44 ± 0.13^e^	5.21 ± 0.15^e^
	90	0.13 ± 0.04^a^	0.92 ± 0.16^b^	9.37 ± 0.17^b^
T_4_	0	0.10 ± 0.01^b^	0.22 ± 0.03^f^	4.51 ± 0.16^f^
	45	0.11 ± 0.01^b^	0.48 ± 0.09^e^	5.36 ± 0.27^e^
	90	0.13 ± 0.03^a^	1.14 ± 0.11^a^	9.66 ± 0.19^a^

In rows and columns, if the means have same letter that are statistically significant (p < 0.05)

FFA: Free Fatty Acids

PV: Peroxide Value

**Table 6:** Sensory characteristics of butter supplemented with microencapsulated chia oil

Treatments	Storage Days	Color	Oxidized Flavor	Texture
Control	0	8.1 ± 0.15	8.1 ± 0.36	8.0 ± 0.20
	45	8.0 ± 0.12	8.0 ± 0.29	7.9 ± 0.31
	90	7.8 ± 0.08	7.9 ± 0.25	7.8 ± 0.07
T_1_	0	8.2 ± 0.19	8.1 ± 0.23	8.1 ± 0.03
	45	8.0 ± 0.17	8.0 ± 0.09	8.0 ± 0.12
	90	8.1 ± 0.13	7.9 ± 0.04	8.0 ± 0.27
T_2_	0	8.1 ± 0.22	7.9 ± 0.42	8.0 ± 0.21
	45	8.0 ± 0.06	7.8 ± 0.17	8.0 ± 0.16
	90	7.9 ± 0.11	7.7 ± 0.10	7.8 ± 0.08
T_3_	0	7.9 ± 0.19	8.0 ± 0.15	8.2 ± 0.19
	45	7.8 ± 0.14	8.1 ± 0.16	8.1 ± 0.15
	90	7.7 ± 0.12	7.8 ± 0.09	8.0 ± 0.26
T_4_	0	8.0 ± 0.05	8.1 ± 0.07	8.1 ± 0.28
	45	7.9 ± 0.16	8.0 ± 0.06	8.1 ± 0.35
	90	7.8 ± 0.25	8.0 ± 0.02	8.0 ± 0.33

All the means mentioned in Table 6 are statistically non-significant (*p* > 0.05)

## Discussion

Results regarding chemical characteristics of chia oil obtained in this investigation are not different from the findings [29]. Fat, moisture and NFDMC of the Ω-3 supplemented butter was within the standards of identity of International Dairy Federation. Several studies have been performed to raise the functional value of butter. Addition of microcapsules of fish oil to yoghurt had no effect on compositional attributes of yoghurt till four weeks of storage [30]. Chemical composition of yoghurt added with flaxseed oil emulsion remained unchanged up to 8 days of storage [31]. In addition to higher magnitude of ALA, chia oil is also known to have superb antioxidant properties. Results of earlier investigation have shown that supplementation of chia oil in cheddar cheese raised the antioxidant properties; oxidative stability of chia oil supplemented cheddar cheese was not less than the sample available in the market [11, 32]. The effect of spray drying on antioxidant characteristics of microencapsulated chia oil was not reported in literature, in this investigation, it was planned to determine the effect of spray drying temperature on antioxidant characteristics of microencapsulated chia oil. The superior antioxidant activity microcapsules of chia oil during the storage duration of 90 days may be connected to the presence of strong antioxidant systems and phenolic compounds present in chia oil and protection provided by the microencapsulating material chitosan. Effect of supplementing microcapsules of chia oil on antioxidant characteristics of butter was also determined. Antioxidant characteristics of butter supplemented with microcapsules of chia oil were due to two factors i.e. indigenous antioxidant system of butter which was comprised of vitamin A, E, D, beta-carotene and lecithin etc. exogenous antioxidant system which was introduced in butter by chia oil. Chia oil is a good source of phenolic compounds, chlorogenic acid, caffeic acid, phenolic glycoside-k and phenolic glycoside-Q are the major phenolic compounds present in chia oil [33]. Milk and dairy products are good source of natural antioxidants [34]. Boiling and pasteurization modes of heat treatments had no effect on antioxidant characteristics of milk for the duration of three days [35]. The superior oxidative stability of chia oil in complex food systems is documented in literature. Antioxidant characteristics of ice cream supplemented with olein fraction of chia oil were not influenced by the storage duration [17]. Hussein et al. [36] studied the effect of microencapsulation on antioxidant characteristics of fennel and cumin oil, microencapsulation significantly decreased the antioxidant capacity of these oils.

It was projected that high temperature of spray drying may lead to cause oxidation of PUFAs and modification of fatty acid profile. During the dehydration process, the condensed wall structures keep the temperature of oil less than 100 °C by preventing the heat transfer [37]. The microcapsules designed using chitosan as a coating material and spray drying for the dehydration had a thermo-protective effect. Due to rapid encapsulation in coating material, alteration in the fatty acid profile could not take place [37]. Goyal et al. [38] found that fatty acid profile of free and microencapsulated flaxseed oil was not different form each other. Quispe-Condori et al. [39] reported that microencapsulation and spray drying had no effect on significant effect on fatty acid profile. During the storage phase, polyunsaturated fatty acids with a greater number of double bonds and longer chain moved towards the surface of microcapsules that increases the rate of oxidation. The alteration in fatty acid profile of microencapsulated fish oil, flaxseed oil was due to the movement of polyunsaturated fatty acids towards the surface of microcapsules [40]. Flaxseed is regarded as the richest sources of ALA; however, the magnitude of ALA in chia oil was more than flaxseed oil. As compared to C_18:1_, ALA (C_18:3_) is about times more vulnerable to oxidation [41]. DHA was not detected in control sample. Fish oil contains about 14–15% EPA and DHA, in current investigation, MCO had more than 73% ALA. The effect supplementation of Ω-3 capsules in bread, fatty acid profile of microcapsules was not different from the free oil. According to the recommendations of ICMR [42] 1.6 g ALA should be consumed on daily basis. Chia oil can is susceptible to oxidation, to overcome the problems of low oxidative stability and development of oxidized flavor, in current investigation chia oil was transformed to microcapsules so that PUFAs can be safeguarded from oxidation. Changes in fatty acid profile provides useful information regarding the oxidative stability of fats and oils [43]. Changes in the fatty acid profile of butter were strongly connected to the oxidation products [44]. Minimum changes were recorded in fatty acid profile of MCO supplemented butter at different stages of storage. Ω-3 fatty acids have less resistance to auto-oxidation, microencapsulation significantly enhanced the oxidative stability of Ω-3 fatty acids. Gonzalez et al. [45] observed that presence of PUFAs in butter decreased its oxidative stability. Dairy products with modified fatty acid profile were more prone to auto-oxidation [46]. Foods added with Ω-3 fatty acids are usually safeguarded from auto-oxidation by antioxidants, in current investigation, microcapsules from chia oil were developed for the supplementation of butter, this may open a new array of functional foods supplemented with Ω-3 fatty acid of chia oil. However, detailed research is required for the exploration of new avenues for the fortification of foods with microcapsules of chia oil. Goyal et al. [38] studied the effect of storage on ALA content of microencapsulated flaxseed oil; it was observed that storage significantly decreased the concentration of ALA. The oxidative stability of ice cream supplemented with microcapsules of flaxseed oil was studied, 21.38% ALA was oxidized after 60 days of storage. A study was conducted to determine the effect of storage time on Ω-3 fatty acids in yoghurt, after 4 weeks of storage, concentration of Ω-3 fatty acids decreased by 14.52%.

Peroxide value quantifies the extent of peroxides in fats and oils; it is an empirical method to anticipate the storage stability of fats and oils. At industrial level, peroxide value is used to estimate the shelf life of fats and oils and other derived foods, foods which have lower peroxide value can be efficiently stored for longer periods of time [47]. Primary oxidation products such as, hydroperoxides have no color and odor but these are highly toxic and hinder in the bioavailability of fatty acids. It is reported in literature that rate of oxidation of ALA is 20 times faster than oleic acid [41]. Chen et al. [48] prepared dairy products from the milk having a greater number of PUFAs, dairy products revealed lower shelf stability as compared to the normal dairy products. Ajmal et al. [49] described that dairy products containing a greater number of PUFAs had a shorter shelf life. Maximum peroxide value recorded in present study was well below than the allowable limit (5 MeqO_2_/kg) given in the CAC [50]. Microcapsules of fish oil in vacuum packaging had superior oxidative stability [51]. Magnitude of conjugated dienes increased when microencapsulated fish oil was stored for the longer time [52]. Free fatty acids are the products of hydrolytic rancidity having no connection with degree of unsaturation. Free fatty acids content of whey butter was analyzed at different stages of storage and free fatty acids in whey butter increased with the advancement of storage [53]. Free fatty acids are highly important from the keeping quality perspectives; they can not only induce bad odors in foods but also can accelerate the auto-oxidation [54]. This research work provided evidence that free fatty acids in microencapsulated chia oil were not increased during the storage and supplemented butter had lower concentrations of free fatty acids. Anisidine value is extensively used for the determination of secondary oxidation products such as non-volatile carbonyl compounds produced during the oxidative degradation of lipids. Under acidic conditions, 2-alkenals and 2,4-dienals react with *p*-anisidine. The rise in anisidine value will mean greater amounts of aldehydes which reflect lower oxidative stability [55]. Anisidine value of good quality edible oils and fats should be less than two [56]. Anisidine value of microencapsulated avocado oil was more resistant to oxidation than free oil [40]. Microencapsulated olive oil had lower anisidine value than crude olive oil [57]. Anisidine value below 10 is usually regarded ideal for the better storage stability of fat rich foods [43]. Rancimat method is based on the measurement of electrical conductivity of distilled water triggered by the production of lipid oxidation compounds, when fats and oils are oxidized in accelerated conditions i.e. higher temperature and aeration [58]. Induction period is directly connected with antioxidant activity and oxidative stability. The non-significant effect on induction period of all the experimental samples can be attributed to microencapsulation. For the estimation of expected shelf life of dairy products, induction period is usually not used, however, in our previous investigation regarding the oxidative stability of olein based butter, we used induction period [54]. Strong correlations were established between peroxide value and induction period, testing intervals showing higher peroxide revealed lower peroxide value. For the estimation of oxidation resistance of whey butter, Boselli et al. [54] used Rancimat method. Barroso et al. [59] compared the induction period of bulk and microencapsulated flaxseed oil, the latter had significantly higher induction period than the former. Induction period of microencapsulated fish oil and sunflower oil was significantly higher than free oils [60]. Similar results were reported about the oxidative stability of olive oil [61].

Sensory characteristics of fat rich dairy products usually decline due to the oxidation of PUFAs. Non-significant difference in color and flavor score may be attributed to the better oxidative stability of microcapsules. Sensory characteristics of cookies added with microencapsulated fish oil were comparable to the cookies without addition of microencapsulated fish oil [62]. de-Conto et al. [63] studied the effect of encapsulated Ω-3 powder on sensory properties of pan bread and addition of Ω-3 powder did not induce undesirable sensory attributes in fortified bread.

## Conclusion

Microcapsules of chia oil were developed for the first time and microencapsulation considerably improved the oxidative stability of Ω-3 fatty acids of chia oil. Supplementation of butter with microencapsulated chia oil at 2, 4, 6 and 8% concentrations significantly enhanced Ω-3 fatty acids in butter. Fatty acid profile of MCO supplemented butter before and after the storage period of 90 days was almost similar. MCO at 8% concentration can be used for raising the amount of Ω-3 fatty acids in butter.

## Data Availability

The dataset supporting the conclusions of this article is included within the article.

## Abbreviations

ALA: Alpha-linolenic acid
DHA: Docosahexaenoic acid
DPA: Docosapentaenoic acid
DPPH: 2, 2-Diphenyl-1-picrylhydrazyle
EPA: Eicosapentaenoic acid
MC: Moisture content
MCO: Microcapsules of chia oil
NFDMC: Non-fat dry matter content
PUFAs: Polyunsaturated fatty acids
